# 2513: Enhancing KL2 Scholar poster communication skills for lay audiences using community judges

**DOI:** 10.1017/cts.2017.183

**Published:** 2018-05-10

**Authors:** Michelle Lamere, Angela Merrifield, Deborah Hendricks, Megan Hoffman, Megan Larson, Sandra Wells, David H. Ingbar

**Affiliations:** CTSI, University of Minnesota, Minneapolis, MN, USA

## Abstract

OBJECTIVES/SPECIFIC AIMS: The 2 primary objectives were to (i) insure that Scholars can effectively communicate the translational impact of their research to a lay audience and (ii) assess the benefits and efficacy of having community, as well as faculty members, judge the translational impact of KL2 Scholar’s poster presentations. An explicit secondary goal was to further the engagement of community members in CTSI-sponsored translational research. METHODS/STUDY POPULATION: CTSI’s Education, Community Engagement, Discovery and Translation, and Translational Workforce Development Cores created the translational impact questions and evaluation sheets. The Community Engagement and Office of Discovery and Translation recruited community judges from their respective networks and they were assigned to relevant studies. Scholars were provided with the judges scoring template in advance. After the Research Poster Session, the KL2 Scholars evaluated the quality of their presentations and the impact of having feedback from Community Judges. The Community Judges evaluated their perceived “added value” to the research presentations and their interactions with the Scholars. Both Scholars and judges completed evaluations of the poster presentation and judging process, performed on a 5-point Likert scale. RESULTS/ANTICIPATED RESULTS: KL2 Scholars felt that the community impact judges provided valuable feedback on their research (3.8/5) and were satisfied overall with the poster session (3.4/5). In evaluating their own presentations, Scholars tended to rate themselves higher (4.2–4.6/5) on the clarity of their translational impact presentations than the community judges rated the Scholars (4.1–4.2/5). Scholars also rated themselves somewhat higher in the quality of their dealing with any ethical issues and their dissemination plan (4.0/5) than the community judges (3.8/5). Judges were very positive and felt they brought value to the experience (4.2–4.4/5). DISCUSSION/SIGNIFICANCE OF IMPACT: Community judges added qualitative value to the Scholar presentations based on the Scholar and community judge evaluations and based on comparison based on prior year poster sessions. Documenting the degree of impact of the combination of this proscribed poster format and community-judging process awaits future assessment of Scholar presentations before and after the next annual poster presentation.

